# Asymptomatic Presentation of Yellow Oleander Poisoning in a 57-Year-Old Saudi Woman: A Case Report

**DOI:** 10.7759/cureus.50317

**Published:** 2023-12-11

**Authors:** Halah R AlMuhaidib, Noof Alabdulwahab, Shaikhah Al-Otaibi, Rima Aldakheel, Dunya Alfaraj

**Affiliations:** 1 Family Medicine, Imam Abdulrahman Bin Faisal University, Dammam, SAU; 2 Emergency Medicine, King Fahad University Hospital, Khobar, SAU; 3 Pediatrics, Imam Abdulrahman Bin Faisal University, Dammam, SAU

**Keywords:** digoxin, plants, environmental toxicology, yellow oleander, thevetia peruviana

## Abstract

Yellow oleander (*Thevetia peruviana*), known for its cardiac glycosides, can cause severe poisoning with varied clinical manifestations, primarily affecting the cardiovascular system. We present a unique case of a 57-year-old Saudi woman with a history of type 2 diabetes, dyslipidemia, and previous meningioma excision who ingested 3.4 grams of yellow oleander fruit, mistaking it for an edible fruit. Remarkably, she remained asymptomatic with no gastrointestinal, neurological, or cardiovascular symptoms. Examination and investigations, including electrocardiograms and laboratory tests, showed no abnormalities. Despite the known high toxicity of yellow oleander and its documented fatal cases, our patient's asymptomatic presentation is rare. This case highlights the importance of close monitoring and observation in yellow oleander ingestion cases, even in the absence of symptoms, due to variable absorption kinetics and potential delayed onset of toxicity. Our findings also underscore the need for public health awareness regarding the identification and dangers of toxic plants like yellow oleander, especially as they are commonly grown at home.

## Introduction

Yellow oleander, a plant containing cardiac glycosides in all parts with varying concentrations, is particularly potent in its seeds [1]. These glycosides bind to the cardiac cells' sodium/potassium adenosine triphosphatase (ATPase) pump, leading to the pump's inactivation. This inactivation causes an increase in intracellular sodium, subsequently impacting the sodium/calcium exchange channels. This process elevates calcium levels in the cells, enhancing myocardial contractility and automaticity [2].

Ingestion of yellow oleander can lead to poisoning. The pharmacokinetics of this poisoning vary based on the concentration of cardiac glycosides in the ingested plant part and the time elapsed since ingestion [3]. The toxicity severity depends on multiple factors, including the form of ingested seeds (e.g., crushed seeds are more toxic), the extent of gastrointestinal absorption, post-ingestion vomiting, and the individual's overall health and existing comorbidities [4].

Clinical features following yellow oleander ingestion include gastrointestinal symptoms like nausea, vomiting, abdominal pain, and diarrhea and neurological symptoms such as weakness, fatigue, confusion, headache, and dizziness. The most critical symptoms are cardiovascular, including arrhythmias, atrioventricular block, atrial and ventricular fibrillation, and hyperkalemia [4]. Asymptomatic cases post ingestion are rare, with limited case reports in the literature. We describe a 57-year-old Saudi woman who presented to our emergency department asymptomatic after ingesting yellow oleander fruit and was subsequently discharged in good health.

## Case presentation

**Figure 1:**
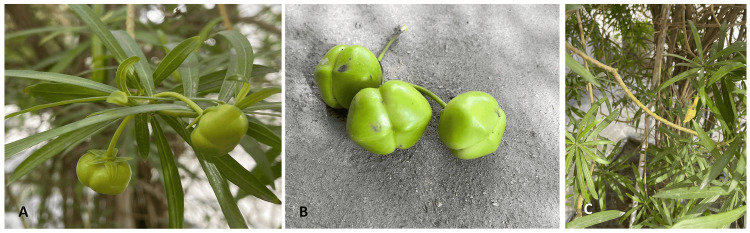
(A) The fruit of yellow oleander. (B) A close view of the yellow oleander's fruit. (C) The tree of yellow oleander, showing the yellow flower Image Credit: Patient

A 57-year-old Saudi woman with a 10-year history of type 2 diabetes managed with metformin, dyslipidemia treated with atorvastatin, and a history of meningioma excision three years prior presented to the emergency department five hours post ingestion of a home-grown yellow oleander fruit. The ingested fruit, excluding seeds, weighed 3.4 grams. She had mistaken it for an edible fruit (Figure 1). The patient exhibited no symptoms; there was no abdominal pain, vomiting, nausea, or bowel habit changes. She also did not experience shortness of breath, chest pain, palpitations, syncope, headache, photophobia, or blurred vision.

**Figure 2:**
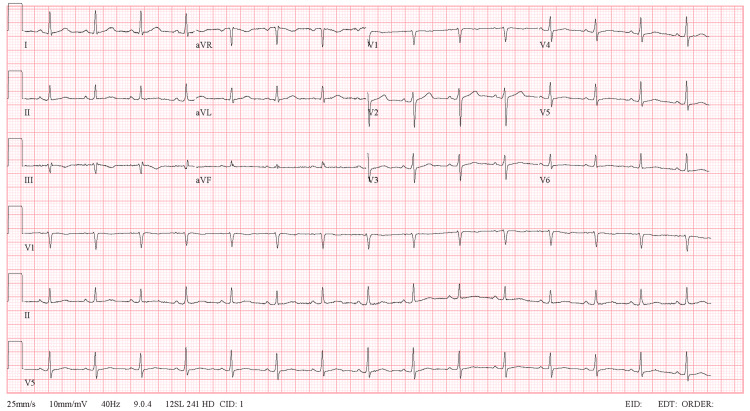
Patient ECG showing normal sinus rhythm. The cardiac axis is normal, P waves are present with normal morphology, there are no pathological Q waves, and the QRS complexes appear normal and narrow ECG: electrocardiogram

Upon examination, the patient was conscious, alert, and oriented, showing no signs of pain or distress. Her vital signs were as follows: blood pressure: 150/90 mmHg, heart rate: 96 beats per minute (bpm), respiratory rate: 20, and oxygen saturation: 99% on ambient air. Cardiovascular examination revealed normal first and second heart sounds with no additional sounds or murmurs. Pulmonary assessment showed clear lungs with bilateral air entry and no added sounds. Abdominal examination indicated a soft, lax, non-tender abdomen with no signs of lower limb edema or deep vein thrombosis. An electrocardiogram (ECG) displayed a normal sinus rhythm with regular intervals (Figure 2). Laboratory test results were normal, including electrolyte, renal, and liver function (Table 1). Digoxin levels were within reference limits.

**Table 1:** Laboratory investigations of the patient pCO2: partial pressure of carbon dioxide; pO2: partial pressure of oxygen

Investigation	Result	Reference range
Arterial blood gas		
pH	7.3	7.35-7.45
pCO2	47.3 mmHg	35-45 mmHg
pO2	41.3 mmHg	75-100 mmHg
Complete blood count		
Hemoglobin	13 g/dL	13-18 g/dL
Hematocrit	34.5%	42-52%
Liver function test		
Alanine aminotransferase	24 U/L	5-34 U/L
Aspartate aminotransferase	37 U/L	5-55 U/L
Digoxin II	<0.19 mg/mL	0.8-2 mg/mL
Renal function test		
Serum urea nitrogen	13 mg/dL	7.0-26 mg/dL
Creatinine	0.65 mg/dL	0.6-1.3 mg/dL
Phosphorus	3.9 mg/dL	2.3-4.7 mg/dL
Magnesium	1.85 mg/dL	1.6-2.6 mg/dL
Calcium	9.4 mg/dL	8.4-10.2 mg/dL
Fasting glucose	138 mg/dL	70-140 mg/dL

The patient was admitted for observation with serial ECGs, venous blood gas, electrolyte panels, and digoxin level monitoring. After 24 hours, she remained asymptomatic, with all laboratory tests showing negative results; serial ECGs confirmed a normal sinus rhythm with regular intervals. All serial digoxin levels were below 0.19 n/ml (reference range: 0.8-2.0 ng/ml). Post discharge, the patient was advised to monitor her heart rate at home at 48 hours, 72 hours, and four days after ingestion, which remained within the reference range of 90-99 bpm.

## Discussion

*Thevetia peruviana*, commonly known as yellow oleander, contains cardiac glycosides that induce digoxin-like toxicity. All parts of the tree, including flowers, leaves, and fruits, are poisonous, with the seeds and roots being the most toxic [5]. Ingestion of these parts can be fatal. It is crucial to monitor patients closely for 24 hours, even without toxicity symptoms, due to variable absorption kinetics. Continuous ECG monitoring is vital for detecting arrhythmias [6].

According to the literature, consuming two to three fruits can cause nausea, vomiting, diarrhea, and lethargy [7]. However, it does not specify the quantity of fruit that leads to cardiac toxicity. Ingestion of more than five crushed seeds is associated with significant toxicity, and the lethal dose ranges from eight to 10 seeds [8]. Remarkably, one seed is equivalent to 100 digoxin tablets [5]. The lethal dose of oleander leaf is approximately 4 grams [9]. Cardiotoxicity from yellow oleander ingestion correlates with higher cardiac glycoside concentrations and serum potassium levels, as hyperkalemia worsens cardiac glycoside toxicity [10,11].

Yellow oleander intoxication presents with symptoms ranging from mild abdominal pain and diarrhea to severe cardiac issues [1]. Most reported cases are symptomatic; however, in our case, the patient was asymptomatic throughout her admission. This is consistent with González-Stuart and Rivera, who reported that 52% of their study sample were asymptomatic [8]. Our report appears to be the only case of asymptomatic intoxication.

Mechanism of toxicity

Cardiac glycosides, by binding to the sodium/potassium ATPase pump in cardiac cells, increase intracellular calcium concentration, thus enhancing myocardial contractility and automaticity [2]. This action may cause various ECG changes following intoxication, such as sinus bradycardia, atrioventricular block, ventricular arrhythmia, T-wave abnormalities, PR segment lengthening, ST interval abnormalities, and absent P waves [12]. However, our patient showed no ECG changes on admission or discharge, aligning with Karthik et al.'s findings that 50% of their study subjects displayed no ECG changes [11]. Cardiac glycosides also inhibit sodium/potassium ATPase in skeletal muscle cells, leading to extracellular potassium accumulation and hyperkalemia [13].

Management

For asymptomatic patients, supportive care is primary. Monitoring hydration status and correcting electrolyte imbalances are essential. Single-dose activated charcoal (SDAC) is effective for gastric decontamination within one to two hours of ingestion and binds cardiac glycosides, as shown in animal studies [6]. Multidose-activated charcoal (MDAC) prevents absorption and reabsorption from enterohepatic circulation and enhances gastric elimination. Our patient did not receive SDAC due to the delayed presentation (five hours post ingestion) or MDAC as she remained asymptomatic.

In cases where symptoms persist, digoxin-specific antibody fragments (Fab) administration is recommended, with an initial dose of at least 800 mg, higher than the usual 400 mg for digoxin toxicity. This higher dose is due to the lower affinity of Fab for natural cardiac glycosides in oleanders [14]. Atropine may be used for bradycardia [13]. Clinical assessment is key; asymptomatic patients who are hemodynamically stable, appear well, and have a normal ECG 24 hours post ingestion can likely be safely discharged [6]. In our case, the patient underwent close observation with serial ECGs, digoxin level testing, and electrolyte monitoring, all of which remained within reference limits.

## Conclusions

Despite its high toxicity, yellow oleander is commonly grown at home, and people may mistakenly consume it, thinking it is edible. Public health awareness about plant identification and its toxicity needs to be raised. Our patient, who ingested one yellow oleander fruit (equivalent to 3.4 grams without seeds), presented asymptomatically and was discharged in good health. Further research is necessary to determine the toxic dose of yellow oleander fruit, as relying on fruit number is unreliable due to size variations.
